# External validation of the intensive care national audit & research centre (ICNARC) risk prediction model in critical care units in Scotland

**DOI:** 10.1186/1471-2253-14-116

**Published:** 2014-12-15

**Authors:** David A Harrison, Nazir I Lone, Catriona Haddow, Moranne MacGillivray, Angela Khan, Brian Cook, Kathryn M Rowan

**Affiliations:** Intensive Care National Audit & Research Centre (ICNARC), Napier House, 24 High Holborn, London, WC1V 6AZ UK; Scottish Intensive Care Society Audit Group, Information Services Division, NHS National Services Scotland, 1 South Gyle Crescent, Edinburgh, EH12 9EB UK; Directorate of Critical Care, Royal Infirmary of Edinburgh, 51 Little France Crescent, Edinburgh, EH16 5SA UK; Centre for Population Health Sciences, University of Edinburgh, Medical School, Teviot Place, Edinburgh, EH8 9AG UK

**Keywords:** Critical care, Intensive care units, Models, Statistical, Prognosis, Risk adjustment, Severity of illness index, Validation studies

## Abstract

**Background:**

Risk prediction models are used in critical care for risk stratification, summarising and communicating risk, supporting clinical decision-making and benchmarking performance. However, they require validation before they can be used with confidence, ideally using independently collected data from a different source to that used to develop the model. The aim of this study was to validate the Intensive Care National Audit & Research Centre (ICNARC) model using independently collected data from critical care units in Scotland.

**Methods:**

Data were extracted from the Scottish Intensive Care Society Audit Group (SICSAG) database for the years 2007 to 2009. Recoding and mapping of variables was performed, as required, to apply the ICNARC model (2009 recalibration) to the SICSAG data using standard computer algorithms. The performance of the ICNARC model was assessed for discrimination, calibration and overall fit and compared with that of the Acute Physiology And Chronic Health Evaluation (APACHE) II model.

**Results:**

There were 29,626 admissions to 24 adult, general critical care units in Scotland between 1 January 2007 and 31 December 2009. After exclusions, 23,269 admissions were included in the analysis. The ICNARC model outperformed APACHE II on measures of discrimination (c index 0.848 versus 0.806), calibration (Hosmer-Lemeshow chi-squared statistic 18.8 versus 214) and overall fit (Brier’s score 0.140 versus 0.157; Shapiro’s R 0.652 versus 0.621). Model performance was consistent across the three years studied.

**Conclusions:**

The ICNARC model performed well when validated in an external population to that in which it was developed, using independently collected data.

**Electronic supplementary material:**

The online version of this article (doi:10.1186/1471-2253-14-116) contains supplementary material, which is available to authorized users.

## Background

Risk prediction models (also termed prognostic models, outcome prediction models or mortality prediction models) are used in critical care for summarising and communicating risk, supporting clinical decision-making and benchmarking performance of health care providers [1]. They can be used in randomised controlled trials for risk stratification and to increase power in adjusted analyses [2], and for risk adjustment in non-randomised comparisons [3]. However, even when developed using robust statistical methods in large, representative data sources, risk prediction models require validation before they can be used with confidence [4]. Ideally, external validation should be conducted using independently collected data from a different source to that used to develop the original model [5].

The Case Mix Programme is the national clinical audit of adult critical care in England, Wales and Northern Ireland. Risk prediction, using an up-to-date, validated model, is essential to underpin benchmarking and comparative reporting. A head-to-head comparison of the most recent versions of all major critical care risk prediction models using data from the Case Mix Programme demonstrated little difference in performance between the models, but with scope for further improvement [6]. The Intensive Care National Audit & Research Centre (ICNARC) risk prediction model was therefore developed and validated using data from the Case Mix Programme with the objective of improving on the existing models [7]. It has subsequently been validated using further data from the Case Mix Programme, including external validation among critical care units that joined the programme after the development of the model [8] but it has never undergone validation using independently collected data.

Scotland is a devolved nation of the United Kingdom (UK) and has a very similar health care system to the rest of the UK. However, it has a separate, independent, national clinical audit for adult critical care, coordinated by the Scottish Intensive Care Society Audit Group (SICSAG) through the Information Services Division of NHS National Services Scotland. Our aim, therefore, was to validate the ICNARC risk prediction model using data from adult, general critical care units in Scotland.

## Methods

### The Scottish intensive care society audit group (SICSAG) database

SICSAG has maintained a national database of patients admitted to adult, general critical care units in Scotland since 1995. Currently, all adult, general and specialist intensive care and combined intensive care/high dependency units (critical care units) in Scotland participate voluntarily in the audit. Data are collected prospectively using a dedicated software system. Annual data extracts are pooled centrally onto servers at the Information Services Division and validation queries relating to discharges, outcomes, ages and missing treatment information are then issued and fed back to individual units for checking by local and regional audit coordinators.

This study was approved by the Privacy Advisory Committee, NHS National Services Scotland (application number 53/10).

### Inclusion and exclusion criteria

Data were extracted from the SICSAG database for all admissions to all 24 adult, general critical care units in Scotland between 1 January 2007 and 31 December 2009. During the study period, specialist cardiothoracic critical care units were not participating in the national audit; admissions to one specialist neurocritical care unit were not included in the data extract. The following admissions were excluded from the analysis: admissions flagged in the database as ‘Exclude from severity of illness scoring’; readmissions of the same patient within the same acute hospital stay; admissions missing the outcome of acute hospital mortality; admissions missing age, location prior to admission or primary reason for admission to the critical care unit; and admissions for whom the primary reason for admission was unable to be mapped onto the ICNARC Coding Method (see below).

### The ICNARC model

The ICNARC model was developed and validated using data from the ICNARC Case Mix Programme [7, 8]. Risk predictions are calculated for each admission based on the following predictors:

-  age in years at admission to the critical care unit;
-  location prior to admission to the critical care unit and urgency of surgery;
-  cardiopulmonary resuscitation within 24 hours prior to admission to the critical care unit;
-  ICNARC Physiology Score – an integer score between 0 and 100 based on derangement in 12 physiological parameters during the first 24 hours following admission to the critical care unit;
-  primary reason for admission to the critical care unit; and
-  interactions between the ICNARC Physiology Score and primary reason for admission.

The ICNARC model is regularly recalibrated to Case Mix Programme data to ensure accurate, contemporaneous comparative audit for the Case Mix Programme. The most appropriate recalibration was selected based on the time period of data included in the analysis – this was a recalibration undertaken in 2009 using Case Mix Programme data from 194,892 admissions to 187 critical care units between 1 January 2006 and 31 December 2008.

In order to apply the ICNARC model to data from the SICSAG database, certain assumptions and recoding were required, detailed below. After applying this recoding, the predicted risk of acute hospital mortality from the ICNARC model was calculated for each admission using standard algorithms developed for the Case Mix Programme.

#### Location prior to admission

In the ICNARC model, for admissions to the critical care unit from an imaging department and those from the recovery area (not for postoperative use but when used as a temporary critical care area), the previous location is used to assign a weight. For admissions collected to Version 0 of the SICSAG dataset (phased out from June 2008 to May 2009), only a single location immediately prior to the critical care unit was recorded and therefore the weightings for location prior to admission for these admissions was assigned based on the most common previous location in both SICSAG Version 203 data (introduced from June 2008) and Case Mix Programme data. Admissions from an imaging department were assumed to have previously been in an emergency department and admissions from the recovery area were assumed to have previously been on a general ward.

#### Systolic blood pressure

In the ICNARC Physiology Score, weighting of the systolic blood pressure (SBP) is based on the lowest value during the first 24 hours following admission to the critical care unit. For SICSAG data (all Versions), only the highest SBP with paired diastolic blood pressure (DBP) and the lowest DBP with paired SBP were recorded. The lowest SBP was therefore imputed using a regression model fitted to 574,864 admissions to 181 critical care units in the Case Mix Programme between 1995 and 2008 with all these parameters recorded. The resulting equation was:


#### Arterial pH

In the ICNARC Physiology Score, weighting of arterial pH is based on the lowest pH during the first 24 hours following admission to the critical care unit. For SICSAG data (all Versions), only the pH from the arterial blood gas with the lowest partial pressure of oxygen (PaO_2_) was recorded. The lowest pH was therefore imputed using a regression model fitted to 1,011,217 admissions to 224 critical care units in the Case Mix Programme between 1995 and 2013 with both pH measurements recorded. The resulting equation was:


#### Neurological status

In the ICNARC Physiology score, weighting of neurological status is based on either the lowest Glasgow Coma Score during the first 24 hours following admission to the critical care unit (for admissions not sedated during that entire period) or a separate weighting for patients that were sedated or paralysed and sedated during the first 24 hours. For admissions collected to Version 203 of the SICSAG dataset (introduced from June 2008), sedation was not recorded. Admissions were therefore assumed to be sedated if they had no lowest Glasgow Coma Score recorded during the first 24 hours following admission to the critical care unit (this was true for 99% of such admissions in SICSAG Version 0 data).

#### Primary reason for admission

In the ICNARC model, weighting of the primary reason for admission to the critical care unit is based on weightings for conditions/body systems from the ICNARC Coding Method [9]. The ICNARC Coding Method is a five-tier, hierarchical system for coding reasons for admission to critical care that contains 795 individual conditions within a hierarchy of type (surgical or non-surgical), body system, anatomical site, pathological or physiological process and individual condition. Coding to the system tier is sufficient to be able to assign a weight for the ICNARC model, although all admissions in the Case Mix Programme are coded to at least the site tier. For all SICSAG data, the primary reason for admission to the critical care unit was collected using Scottish Intensive Care Society (SICS) diagnostic coding. These diagnoses were mapped to appropriate codes within the ICNARC Coding Method by a consultant intensivist with extensive experience of coding data for the Case Mix Programme. Of the 423 SICS diagnoses in use, 295 (70%) were mapped to a specific condition in the ICNARC Coding Method, 44 (10%) were mapped to the process tier of the hierarchy, 37 (9%) to the site tier, 28 (7%) to the system tier, and 19 (4%) were unable to be mapped (see Additional file 1).

### The APACHE II model

The Acute Physiology And Chronic Health Evaluation (APACHE) II model was selected as a comparator for this study as it was the model in use in Scotland at that time. The SICSAG database does not include all the requisite fields to enable a head-to-head comparison against other, more recent, risk prediction models. The APACHE II model was originally developed using data from 19 critical care units in 13 US hospitals [10], and has subsequently been validated and recalibrated using UK data [6, 11]. Risk predictions are calculated for each admission based on the following predictors:

-  the APACHE II Score – an integer score between 0 and 71 comprising an Acute Physiology Score (0–60 points) based on derangement in 12 physiological parameters during the first 24 hours following admission to the critical care unit, age points (0–6) for age categories of ≤44, 45–54, 55–64, 65–74 or ≥75 years, and chronic health points (0–5) for very severe conditions in the past medical history;
-  admission to the critical care unit following emergency surgery; and
-  diagnostic categories based on the primary reason for admission to the critical care unit.

Values of predicted acute hospital mortality were supplied by the Information Services Division, calculated from the original published coefficients [10] using the standard algorithms applied for routine reporting of the SICSAG audit results at that time.

### Statistical methods

The ICNARC model was validated using measures of calibration, discrimination and overall fit, as described below. The validation was conducted in the full three-year SICSAG database extract and for each year separately.

Discrimination was assessed by the c index [12], which is equivalent to the area under the receiver operating characteristic (ROC) curve [13]. Calibration was assessed graphically and tested using the Hosmer-Lemeshow test for perfect calibration in ten equal sized groups by predicted probability of survival [14]. As the Hosmer-Lemeshow test does not provide a measure of the magnitude of miscalibration and is very sensitive to sample size [15], calibration was also assessed using Cox’s calibration regression, which assesses the degree of linear miscalibration by fitting a logistic regression of observed survival on the predicted log odds of survival from the risk model [16]. Accuracy was assessed by Brier’s score (the mean squared error between outcome and prediction) [17] and Shapiro’s R (the geometric mean of the probability assigned to the event that occurred) [18], and the associated approximate R-squared statistics (termed the ‘sum-of-squares’ R-squared and the ‘entropy-based’ R-squared, respectively), which are obtained by scaling each measure relative to the value achieved from a null model [19].

The performance of the ICNARC model was compared with that of the APACHE II model. The difference in c index between the two models was assessed using the method of DeLong et al. [20]. Confidence intervals for observed acute hospital mortality were calculated using the method of Wilson [21].

All statistical analyses were performed using Stata/SE Version 13.0 (StataCorp LP, College Station, Texas, USA).

## Results

Data were extracted from the SICSAG database for 29,626 admissions to 24 adult, general critical care units between 1 January 2007 and 31 December 2009. The following admissions were excluded: 3,599 admissions (12.1%) flagged in the database as ‘Exclude from severity of illness scoring’ (see Table 1 for breakdown of reasons for exclusion); 1,324 (4.5%) readmissions of the same patient within the same acute hospital stay; 173 (0.6%) admissions missing the outcome of acute hospital mortality; 869 (2.9%) admissions missing location prior to admission (n = 16) or primary reason for admission to the critical care unit (n = 864) – no admissions were missing age; and 392 (1.3%) admissions for whom the primary reason for admission was unable to be mapped. This resulted in a cohort of 23,269 (78.5%) admissions for analysis.

**Table 1:** **Reasons for exclusion**

Reason for exclusion	Number (%)	Acute hospital mortality, Deaths/N (%)
Excluded from APACHE II	445 (1.5)	290/407 (71.3)
*Death within 4 hours*	*231 (0.8)*	*231/231 (100)*
*Missing core physiology data*	*103 (0.3)*	*33/101 (32.7)*
*Age less than 16 years*	*65 (0.2)*	*5/30 (16.7)*
*Admission for primary burn injury*	*46 (0.2)*	*21/45 (46.7)*
Low risk patients	2,305 (7.8)	174/2291 (7.6)
*High dependency unit patient*	*1,707 (5.8)*	*116/1694 (6.8)*
*Admission for post-surgical recovery*	*598 (2.0)*	*58/597 (9.7)*
Responsibility of other team	88 (0.3)	35/88 (39.8)
*Awaiting transfer*	*45 (0.2)*	*22/45 (48.9)*
*In critical care under another team*	*43 (0.1)*	*13/43 (30.2)*
Unspecified	761 (2.6)	232/743 (31.2)
*‘Unit decision not to score patient’*	*369 (1.2)*	*118/360 (32.8)*
*Other (unspecified)*	*298 (1.0)*	*87/293 (29.7)*
*Reason missing or not documented*	*94 (0.3)*	*27/90 (30.0)*

Reasons for exclusion for patients flagged in the SICSAG database extract as ‘Exclude from severity of illness scoring’.

APACHE, Acute Physiology And Chronic Health Evaluation; SICSAG, Scottish Intensive Care Society Audit Group.

Of the admissions flagged as ‘Exclude from severity of illness scoring’, acute hospital mortality was reported for 3,529 admissions (98.1%) and, of these, 731 (20.7%) died before discharge from acute hospital (see Table 1 for breakdown). It was not possible to include these patients in the analysis, even using statistical imputation methods to account for missing data, as insufficient predictor data were recorded. Due to the large number of admissions flagged as ‘Exclude from severity of illness scoring’, a post hoc analysis was undertaken to investigate the potential impact of such exclusions using Case Mix Programme data (see below).

**Figure 1:**
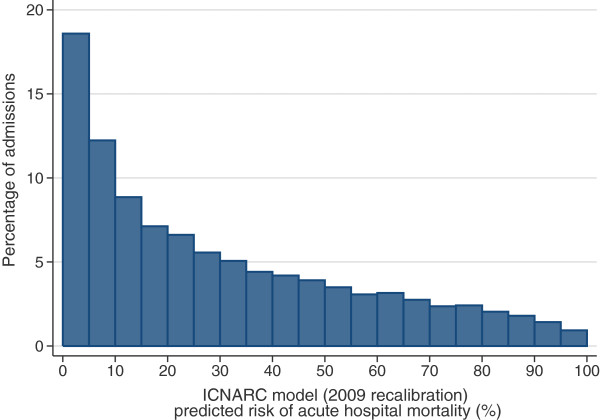
**Distribution of predicted risk.** Distribution of predicted risk from the ICNARC risk prediction model (2009 recalibration) among 23,269 admissions to adult, general critical care units in Scotland.

Table 2 summarises the case mix and outcomes for the included admissions, overall and for each year. The mean age was 57 years, 56% of admissions were male, and two thirds of admissions were non-surgical. These characteristics were relatively stable over the three year period. The distribution of predicted risk of acute hospital death from the ICNARC model (2009 recalibration) is shown in Figure 1. The mean predicted risk of death (expected acute hospital mortality) was 30.1%, which was very close to the overall observed acute hospital mortality of 29.7%.

**Table 2:** **Summary of included admissions**

Characteristic	Overall	2007	2008	2009
Number of admissions	23,269	7,396	7,994	7,879
Age				
Mean (SD)	57.5 (18.0)	57.6 (18.1)	57.4 (18.2)	57.5 (17.8)
Median (IQR)	61 (45, 72)	61 (45, 72)	61 (45, 72)	61 (45, 71)
Sex, n (%)				
Female	10,211 (43.9)	3,218 (43.5)	3,543 (44.3)	3,450 (43.8)
Male	13,058 (56.1)	4,178 (56.5)	4,451 (55.7)	4,429 (56.2)
Surgical status, n (%)				
Elective/scheduled	2,438 (10.5)	695 (9.4)	846 (10.6)	897 (11.4)
Emergency/urgent	5,196 (22.4)	1,580 (21.4)	1,851 (23.2)	1,765 (22.5)
Non-surgical	15,608 (67.2)	5,121 (69.2)	5,296 (66.3)	5,191 (66.1)
ICNARC Physiology Score				
Mean (SD)	19.6 (9.5)	20.0 (9.5)	19.4 (9.5)	19.2 (9.4)
Median (IQR)	18 (12, 25)	18 (13, 26)	18 (12, 25)	18 (12, 25)
ICNARC model (2009 recalibration) predicted risk of acute hospital mortality (%)				
Mean (SD)	30.1 (26.3)	31.2 (26.6)	29.7 (26.3)	29.6 (26.0)
Median (IQR)	22.3 (7.3, 47.9)	24.0 (7.8, 49.6)	21.8 (7.1, 47.0)	21.4 (7.2, 47.3)
APACHE II Score				
Mean (SD)	19.1 (8.1)	19.2 (8.0)	19.1 (8.2)	18.9 (8.2)
Median (IQR)	18 (13, 24)	19 (13, 24)	18 (13, 24)	18 (13, 24)
APACHE II predicted risk of acute hospital mortality (%)				
Mean (SD)	33.0 (25.3)	33.3 (25.0)	32.9 (25.3)	32.8 (25.5)
Median (IQR)	27.4 (11.3, 49.7)	28.5 (12.0, 49.7)	27.0 (11.3, 49.7)	26.6 (10.9, 50.1)
Acute hospital mortality				
Deaths (%)	6,907 (29.7)	2,296 (31.0)	2,342 (29.3)	2,269 (28.8)
[95% CI]	[29.1, 30.3]	[30.0, 32.1]	[28.3, 30.3]	[27.8, 29.8]

Summary of included admissions for the full three-year SICSAG database extract and for each year from 2007 to 2009.

APACHE, Acute Physiology And Chronic Health Evaluation; CI, confidence interval; ICNARC, Intensive Care National Audit & Research Centre; IQR, interquartile range; SD, standard deviation; SICSAG, Scottish Intensive Care Society Audit Group.

**Figure 2:**
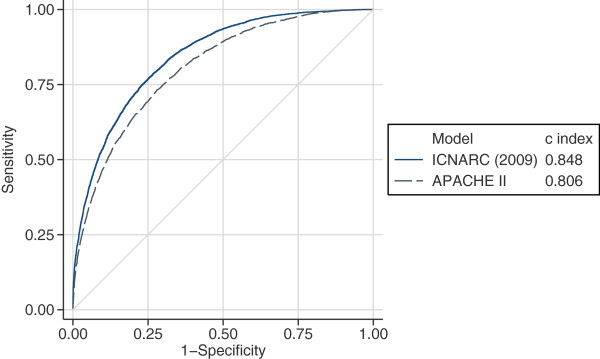
**Receiver operating characteristic curves.** Receiver operating characteristic (ROC) curves for the ICNARC (2009 recalibration) and APACHE II risk prediction models among 23,269 admissions to adult, general critical care units in Scotland.

**Figure 3:**
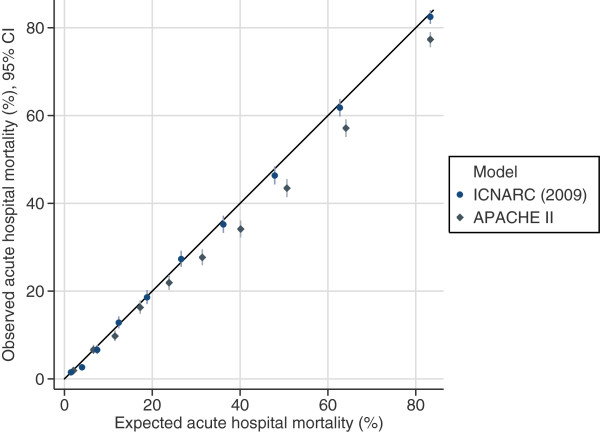
**Calibration plots.** Calibration plots showing observed against expected mortality in ten equal sized groups for the ICNARC (2009 recalibration) and APACHE II risk prediction models among 23,269 admissions to adult, general critical care units in Scotland.

The measures of model performance for the ICNARC model (2009 recalibration), compared with APACHE II, are shown in Table 3. The ICNARC model outperformed APACHE II on all measures of model performance. The ICNARC model had substantially better discrimination (c index 0.848 versus 0.806, P < 0.001, Figure 2) and was also much better calibrated (Figure 3). Cox calibration regression showed an intercept and slope for the ICNARC model very close to the ideal values of 0 and 1, respectively. In contrast, the APACHE II model both underpredicted risk (intercept < 0) and underpredicted variability (slope < 1). Performance of the ICNARC model remained consistent across the three years studied.

**Table 3:** **Measures of model performance**

Measures of model performance	Overall	2007	2008	2009
***ICNARC model***	***N = 23,269***	***N = 7,396***	***N = 7,994***	***N = 7,879***
c index (95% CI)	0.848 (0.843, 0.853)	0.846 (0.837, 0.855)	0.852 (0.843, 0.861)	0.845 (0.836, 0.854)
Hosmer-Lemeshow test				
Chi-squared (P-value)	18.8 (0.043)	3.5 (0.97)	12.7 (0.24)	10.8 (0.37)
Cox calibration regression				
Intercept (95% CI)	-0.02 (-0.06, 0.02)	-0.02 (-0.07, 0.06)	-0.01 (-0.08, 0.06)	-0.05 (-0.12, 0.02)
Slope (95% CI)	1.02 (0.99, 1.05)	1.02 (0.96, 1.07)	1.04 (0.98, 1.09)	1.01 (0.96, 1.06)
Chi-squared (P-value)	5.3 (0.070)	0.5 (0.78)	2.9 (0.24)	3.6 (0.17)
Brier’s score	0.140	0.143	0.137	0.139
Sum-of-squares R^2^	0.331	0.331	0.338	0.325
Shapiro’s R	0.652	0.646	0.656	0.653
Entropy-based R^2^	0.296	0.295	0.303	0.290
***APACHE II***	***N = 22,700***	***N = 7,277***	***N = 7,992***	***N = 7,431***
c index (95% CI)	0.806 (0.800, 0.812)	0.793 (0.782, 0.804)	0.808 (0.798, 0.818)	0.817 (0.807, 0.827)
Hosmer-Lemeshow test				
Chi-squared (P-value)	214 (<0.001)	44.9 (<0.001)	85.1 (<0.001)	120 (<0.001)
Cox calibration regression				
Intercept (95% CI)	-0.26 (-0.30, -0.23)	-0.18 (-0.24, -0.12)	-0.27 (-0.33, -0.21)	-0.34 (-0.40, -0.28)
Slope (95% CI)	0.91 (0.89, 0.94)	0.88 (0.83, 0.93)	0.92 (0.87, 0.97)	0.95 (0.90, 1.00)
Chi-squared (P-value)	208 (<0.001)	39.2 (<0.001)	77.1 (<0.001)	117 (<0.001)
Brier’s score	0.157	0.165	0.156	0.151
Sum-of-squares R^2^	0.244	0.234	0.246	0.250
Shapiro’s R	0.621	0.608	0.623	0.631
Entropy-based R^2^	0.214	0.200	0.217	0.224

Measures of model performance for the ICNARC model (2009 recalibration) compared with the APACHE II model for the full three-year SICSAG database extract and for each year from 2007 to 2009.

APACHE, Acute Physiology And Chronic Health Evaluation; CI, confidence interval; ICNARC, Intensive Care National Audit & Research Centre; SICSAG, Scottish Intensive Care Society Audit Group.

In simulations using Case Mix Programme data to reproduce the potential impact of the exclusion of patients flagged as ‘Exclude from severity of illness scoring’, randomly excluding an equivalent proportion of the same types of patients resulted in the following percentage changes in measures of model performance: c index from -0.3% to +0.02%; Brier’s score from -0.8% to +3.8%; and ratio of observed to expected deaths from -1.1% to +0.6%.

## Discussion

The ICNARC model demonstrated excellent performance when validated in an external sample of data collected from adult, general critical care units in Scotland. The model performance exceeded that of the APACHE II model, being used for benchmarking outcomes in Scotland at the time of this study, on all measures and was consistent over time.

The discrimination of the ICNARC model (c index 0.848) was slightly lower than that reported previously from the original development and validation samples (0.872 and 0.870, respectively) [7] and previous external validation using data from the same source but from different critical care units (0.868) [8]. The finding that all measures of model performance were consistent over time was surprising, as previous studies have suggested that while discrimination of risk models is maintained, calibration deteriorates over time, necessitating regular recalibration of the models [6, 22].

The main strength of this study is the large, representative dataset. As these data come from a very similar healthcare system to the rest of the UK, where the model was developed, but were collected, managed and validated independently, they represent the ideal setting in which to validate the ICNARC model. Independent, external validation of the ICNARC model within the rest of the UK is impossible as the Case Mix Programme has 96% coverage meaning that there are not sufficient critical care units outside of the Case Mix Programme for this to be done.

The study does have some limitations, most notably the number of admissions that it was necessary to exclude. One fifth of exclusions were of multiple admissions of the same patient, which are essential to exclude as outcomes for these admissions are not independent, and follow-up was excellent, with only 0.6% of admissions excluded due to missing outcomes. However, the largest category of exclusions was those flagged as ‘Exclude from severity of illness scoring’ (12.1% of all admissions). The main reason for these exclusions seems to have been to reduce the data collection burden for admissions that would not have been included in benchmarking using the APACHE II model and those considered to have a very low risk of death. However, 761 admissions (2.6% of all admissions) were excluded without any clear reason being specified. The excluded admissions did not have sufficient data recorded to be able to reinstate them into the analysis, however simulating similar exclusions in Case Mix Programme data demonstrated that the impact of these exclusions was likely to be small.

It was necessary to apply some assumptions and mapping of data in order to be able to apply the ICNARC model to the SICSAG dataset. The simplest approach to assigning weights for lowest systolic blood pressure and lowest arterial pH would have been to use the most similar available value of these parameters (the systolic blood pressure associated with the lowest diastolic blood pressure and the pH from the arterial blood gas with the lowest PaO_2_), however, this would have resulted in measurements that were slightly less extreme than the true values and therefore potentially underestimated risk of death. We therefore used data from the Case Mix Programme to develop appropriate regression imputation equations. Following a dataset revision, explicit recording of sedation during the first 24 hours in the critical care unit was removed from the SICSAG dataset. It was therefore necessary to make the assumption that patients with no Glasgow Coma Score recorded were sedated. Using the earlier portion of the dataset, where explicit recording of sedation was available, this assumption was demonstrated to be reasonable, with 99% of missing Glasgow Coma Score values being due to sedation. Any impact on risk predictions will therefore have been minimal.

It was also necessary to map reasons for admission to critical care, which had been recorded using a different coding system. Although only 70% of the diagnostic categories could be mapped to a specific condition in the ICNARC Coding Method, the hierarchical nature of the ICNARC Coding Method enabled most of the remaining diagnostic categories to be mapped to a higher level in the hierarchy, and only 4% of diagnostic categories were unable to be mapped resulting in the exclusion of 1.3% of admissions. It is possible that the slightly less specific diagnostic coding, combined with the need to map these onto a different coding system, may have contributed to the slightly lower discrimination of the ICNARC model than seen in Case Mix Programme data.

## Conclusions

The ICNARC model performed well when validated in an external population to that in which it was developed, using independently collected data. The ICNARC model outperformed APACHE II on measures of discrimination, calibration and overall fit.

## Electronic supplementary material

Additional file 1: **Scottish Intensive Care Society diagnoses that were unable to be mapped to the ICNARC Coding Method.** This file details the 19 diagnoses from the Scottish Intensive Care Society diagnostic coding system that were unable to be mapped to the ICNARC Coding Method. (PDF 74 KB)

## Abbreviations

APACHE: Acute Physiology And Chronic Health Evaluation
DBP: diastolic blood pressure
ICNARC: Intensive Care National Audit & Research Centre
PaO_2_: partial pressure of oxygen
ROC: receiver operating characteristic
SBP: systolic blood pressure
SICSAG: Scottish Intensive Care Society Audit Group
UK: United Kingdom.
