# Supplementary material for: External validation of the intensive care national audit & research centre (ICNARC) risk prediction model in critical care units in Scotland
Source: BMC Anesthesiol. 2014 Dec 15;14:116. doi: 10.1186/1471-2253-14-116 (PMC4277842; doi:10.1186/1471-2253-14-116)
Supplement: Supplementary file 1 — Additional file 1: Scottish Intensive Care Society diagnoses that were unable to be mapped to the ICNARC Coding Method. This file details the 19 diagnoses from the Scottish Intensive Care Society diagnostic coding system that were unable to be mapped to the ICNARC Coding Method. (PDF 74 KB) [file 12871_2014_323_MOESM1_ESM.pdf]

Additional file 1 - Scottish Intensive Care Society diagnoses that were unable to be mapped to the ICNARC Coding Method

- Disseminated malignancy
- Endoscopy
- Interventional radiology
- Interventional radiology/cardiology
- MRSA
- Massive blood loss/transfusion without shock
- Massive blood transfusion
- Multiple surgical procedures
- Other anaesthetic complication
- Other chronic physical disorder
- Other drug related problem
- Other infection
- Other surgery
- Other trauma
- Pre-op assessment/monitoring/optimisation
- Self-inflicted injury
- Surgical complication
- Systemic embolism
- VRE
